# Addressing the Global Burden of Trauma in Major Surgery

**DOI:** 10.3389/fsurg.2015.00043

**Published:** 2015-09-03

**Authors:** Geoffrey P. Dobson

**Affiliations:** ^1^Heart, Trauma and Sepsis Research Laboratory, Australian Institute of Tropical Health and Medicine, College of Medicine and Dentistry, James Cook University, Townsville, QLD, Australia

**Keywords:** perioperative, injury, trauma, surgery, inflammation, coagulopathy, endothelium, sepsis

## Abstract

Despite a technically perfect procedure, surgical stress can determine the success or failure of an operation. Surgical trauma is often referred to as the “neglected step-child” of global health in terms of patient numbers, mortality, morbidity, and costs. A staggering 234 million major surgeries are performed every year, and depending upon country and institution, up to 4% of patients will die before leaving hospital, up to 15% will have serious post-operative morbidity, and 5–15% will be readmitted within 30 days. These percentages equate to around 1000 deaths and 4000 major complications every hour, and it has been estimated that 50% may be preventable. New frontline drugs are urgently required to make major surgery safer for the patient and more predictable for the surgeon. We review the basic physiology of the stress response from neuroendocrine to genomic systems, and discuss the paucity of clinical data supporting the use of statins, beta-adrenergic blockers and calcium-channel blockers. Since cardiac-related complications are the most common, particularly in the elderly, a key strategy would be to improve ventricular-arterial coupling to safeguard the endothelium and maintain tissue oxygenation. Reduced O_2_ supply is associated with glycocalyx shedding, decreased endothelial barrier function, fluid leakage, inflammation, and coagulopathy. A healthy endothelium may prevent these “secondary hit” complications, including possibly immunosuppression. Thus, the four pillars of whole body resynchronization during surgical trauma, and targets for new therapies, are: (1) the CNS, (2) the heart, (3) arterial supply and venous return functions, and (4) the endothelium. This is termed the Central-Cardio-Vascular-Endothelium (CCVE) coupling hypothesis. Since similar sterile injury cascades exist in critical illness, accidental trauma, hemorrhage, cardiac arrest, infection and burns, new drugs that improve CCVE coupling may find wide utility in civilian and military medicine.

As the patient goes to the operating room and anesthesia is induced, trauma is suffered and convalescence begins.

Moore (1) p. 291

## Introduction

### Major surgery, trauma and the stress response

Major surgery is defined “as any intervention in a hospital operating theater that requires incision, excision, manipulation or suturing of tissue occurring and requiring regional or general anesthesia or profound sedation to control pain” (2). Surgical trauma is defined as any injury produced by or related to major surgery. As recognized by surgeon Francis Moore over 60 years ago, trauma begins early in the operating room before general anesthesia and before the first surgical incision (1). The extent of injury will depend on the type and duration of surgery and anesthesia, the presence of cardiopulmonary bypass (CPB), a patient’s age, gender, pre-existing health status, medication profile, fluid loading and post-operative pain (3–6). Age is a particular concern because many older patients present with multiple comorbidities and have decreased reserves to cope with the stress of surgery (7–9) while the very young are often more sensitive to general anesthetics (10).

The stress response can be broadly defined as the body’s response to a “stressor,” which may be an injury, hemorrhage, infection or a burn. It is a physiological reaction of profound importance in nature and medicine. From an evolutionary perspective, the stress response would have been triggered by the central nervous system (CNS) to protect against macroscopic “external” threats (e.g., fight-or-flight response) followed by an underlying “internal” response to promote survival, which included activation of the acute inflammatory or immune systems to initiate wound repair and protect against pathogens (11). Rapid protective mechansisms would have conferred a profound “survival fitness” from events such as escaping predators, hunting accidents, or defending a territory. Although highly protective by design, there are *chinks in the stress response’s “internal” evolutionary armor*. Under certain conditions, such as major surgery, instead of inflammation being self-limiting and restorative, the response can exceed the body’s internal tolerances and become “overexpressed” and lead to further injury. The working hypothesis underpinning this review is that if surgical stress can be halted or diminished, patient outcomes will be improved since most cells in the body are genetically programed in that direction of repair. It is about “helping the body help itself.”

### Objective and scope of the problem

Surgery has recently been referred to as the “neglected stepchild of global health”

Farmer and Kim (12) and Rose et al. (13)

Surgical stress is a global problem. In a recent modeling study, Weiser and colleagues estimated there are around 234 million non-cardiac surgical procedures performed annually around the world (2), 40 million in the USA (14) and 19 million in Europe (15). Of these patients, around 30% have an increased risk of cardiovascular complications, or 70 million patients per year. In the USA, Leape and colleagues recently analyzed nearly 500,000 surgeries and reported a 30-day readmission rate of 5.7%, with over 30% being surgical site infections (16). In another study, Dimick and Ghaferi analyzed nearly 60,000 patients from 112 Department of Veterans Affairs hospitals, and found that the overall 30-day readmission rate was two-times higher (11.9%) than Leape’s study, mostly from higher surgical site infections (56%) (17). These data, and others, led Bartels to write that: “The magnitude of all-cause perioperative mortality would make it the number three cause of death in the USA” (18). Moreover, in 28 European nations, Pearse and colleagues conducted a 7-day cohort study among 46,539 non-cardiac surgery patients from 498 hospitals, and found that 4% of total patients died before hospital discharge, and 8% were admitted to critical care with a median length stay of 1–2 days (19). The group concluded that new strategies are urgently required to improve perioperative outcomes because 73% of these surgical deaths occurred *before admittance* to critical care (19).

Combining the available data, surgery-related deaths range from 0.4 to 4% and post-operative morbidity from 5 to 15% (2, 19). *Thus, up to 9 million patients die each year during or immediately after major surgery, and up to twice this number have post-operative complications*. Since major surgery addresses around 11% of the global burden of disease (20), it can be deduced from the available statistics that: (1) surgical trauma already is in global crisis, (2) the large differences in mortality and morbidity within and across countries and institutions are unacceptable, and (3) around half of the deaths and complications may be preventable. New drug therapies could save up to 500 lives every hour.

## Homeostasis, Trauma and the “Steady-State”

The coordinated physiological processes which maintain most of the steady states in the organism are so complex and so peculiar to living beings - involving, as they may, the brain and nerves, the heart, lungs, kidneys and spleen, all working cooperatively - that I have suggested a special designation for these states, homeostasis.

Cannon (21) p. 20–24

Todays’ understanding of the stress response has its roots firmly embedded in Walter B. Cannon’s concept of homeostasis. Cannon formally introduced the concept in 1926, which he cited in his 1929 review: *Organization for Physiological Homeostasis* (22, 23). Cannon’s homeostasis was built on Pfluger’s concept of “steady-state” (1877), Claude Bernard’s concept of “milieu intérieur” (1878), and Richet’s “stability of the organism” (1900) (22, 24). Cannon argued that a living organism was a system in *dynamic state of constancy*, with its constituent parts and processes being actively maintained in constant balance despite external fluctuations. This “balance” required a continuous exchange of matter and energy between the organism and the environment, and internal regulatory mechanisms to keep it in-check. Thus, Cannon’s homeostasis was not an *equilibrium state*, but a *steady-state*. This distinction is important because all living organisms are open systems that rely on a *net flow of matter and energy with time* whereas a strict equilibrium state, by definition, has no net flux (25). A stress, injury, or sickness was now seen in new light, and viewed as a challenge to the body’s dynamic steady-state. Thus a major goal of any drug therapy or treatment was to restore that balance.

## Early History of the Surgical Stress Response

### George crile and “stress-free” surgery

In the early 1900s, the idea of lowering surgical stress was spearheaded by neurosurgeon George W. Crile (1864–1943) at the Cleveland Clinic (26). Crile’s operative technique to improve recovery was revolutionary and included lightly anesthetizing the patient with mask inhalation of nitrous oxide and oxygen, and infiltrating all tissues with a dilute local anesthetic procaine *before the first incision* (27). General anesthesia was insufficient to reduce the patient’s stress response, and so he proposed the word *anoci-association* (*anoci*; noxious or harmful and *associations*; stimuli) to describe the potentially harmful “stressors” during surgery. For a “shockless” operation, Crile suggested a combination of sedation, local and regional anesthesia to reduce pain and improve recovery (28). Crile’s called his concept “stress-free anesthesia” (29), which formed the basis of modern “preemptive anesthesia” (see Optimizing Anesthesia and Analgesia to Reduce Pain and the Stress Response). Neurosurgeon Harvey Cushing (1869–1939) extended Crile’s revolutionary ideas and promoted the use of regional blocks before removing ether anesthesia from the patient to guarantee an optimal postoperative recovery (30). Cushing also confirmed Crile’s observation that surgical shock could be prevented by the careful monitoring of blood pressure and avoiding the “stressors” associated with surgery (30, 31). These century-old ideas and practices from two giants in medicine form the basis of modern-day anesthesiology.

### Cuthbertson’s “ebb and flow” injury hypothesis and the HPA axis

In the early 1930s, Scottish Chemist David Cuthbertson was among the first to characterize the “stress response to injury” (32). Cannon had already suggested a role for an activated sympathetic nervous system with adrenal secretions to increase cardiac output (CO) and mobilize energy stores in the “fight-or-flight” response (22, 33). However, Cuthbertson found in his patients with long bone fractures a dramatic rise in the loss of nitrogen (as urea), potassium, phosphorus, sulfur, creatine and creatinine compared with volunteers with no injury (32). This was groundbreaking because it suggested that *trauma itself* induced a “stress response.” Cuthbertson divided the body’s response into two quantifiable events: (1) An early “ebb” phase, which began 2 h post-injury and lasted 2–3 days; this was associated with a decrease in CO, reduced tissue perfusion, a lower metabolic rate and glucose intolerance, and: (2) a second “flow” phase lasting days and weeks, that was characterized by an increase in metabolic rate, a hyperdynamic circulation (higher CO, respiratory rate), hyperglycemia, a negative nitrogen balance, and muscle wasting (32, 34). The extent and duration of both “ebb” and “flow” phases depended upon the severity of the injury. Today, whole body energy consumption following major surgery (e.g., abdominal) can increase up to 1.5 times (up to 5 ml O_2_/kg/min) (35).

Over the next few decades, the body’s stress response to injury was identified to be under “neural control via the hypothalamus and the hypophyseal portal vessels of the pituitary stalk” (36). This grouping of the “responses” within the CNS and adrenal glands was termed the hypothalamic–pituitary-adrenal (HPA) axis (37). Cuthbertson lean muscle wasting was now viewed as a CNS-linked-catecholamine response, which could be blunted by beta-adrenergic, but not alpha-adrenergic blockers (3). Today, the HPA axis and catecholamines have many diverse functions from controlling CO and metabolism to selectively regulating the compliance, capacitance and blood volume of the systemic, splanchnic and venous vasculature (38). Within seconds of catecholamine release, nearly two-thirds of the splanchnic blood volume (~800 ml) can be autotransfused into the systemic circulation during times of stress (38). Thus catecholamine surges and changes in blood volume and shifts during surgery may be a potential target to improve patient outcomes following surgery (see Injury, Inflammation and Multiple Organ Failure, The First Incision, Effects of Anesthesia on the Surgical Stress Response, and Effects of Major Surgery on Other Organs) (7–9).

## Injury, Inflammation and Multiple Organ Failure

Another major milestone in unraveling the stress response was the discovery of the relationship between injury, inflammation, infection and organ dysfunction (39). The history of inflammation dates back to the ancient Egyptians and Greeks, and the Roman medical writer Celsus in the first century AD who characterized injury by rubor (redness), tumor (swelling), calor (heat), dolor (pain) and functio lesa (loss of function) (40). Along with advances in immunology and molecular biology in the 1970s, a mechanistic link between injury and inflammation was serendipitously found by surgeon Arthur Baue who noticed a progressive, systemic organ failure in his intensive care patients (41). Two years later, Eiserman and colleagues termed this phenomenon multiple organ failure (MOF) and described it as a “fatal expression of *uncontrolled infection*” (42). Infection, however, was only part of the story.

In the 1980s, a German team led by Faist (43) and a Dutch group led by Goris (44) found that MOF developed in trauma patients, *but curiously these patients did not have an infection*. The new “stressor” was an “autodestructive inflammatory response,” and carried a mortality of over 50% (45). This devastating affliction was termed the *systemic inflammatory response syndrome* (SIRS) and was accompanied with delayed immunosuppression (46). Mild immunosuppression, like mild stress, is generally not harmful but if it progresses, secondary infections may occur followed by late MOF and death (47). Late MOF became so prevalent that Deitch wrote: “MOF has reached epidemic proportions in most intensive care units and is fast becoming the most common cause of death in the surgical intensive care unit” (39). Today, MOF remains the greatest contributor to late-trauma mortality and morbidity than any other cause (47).

Currently, animal studies to investigate MOF and SIRS are based on “one-hit” and “two-hit” models proposed by Ernest and Fred Moore (45, 48). In the “one hit” model, the initial stressor (trauma, hemorrhage, sepsis, or burn) is so massive that the subject is overwhelmed with SIRS and succumbs to MOF (49). In the “two-hit” model, less severe injury slowly develops into MOF from *reactivation* of an earlier “minor” inflammatory response (“secondary-hit”), which may cascade into a life-threatening situation (50). While significant overlap exists, the two models, and other variants, attribute MOF to an unchecked inflammation, cardiac depression, HPA axis activation, sympathetic discharge, coagulopathy, and impairment of mitochondrial function (47, 51). After severe to catastrophic trauma, loss of whole body homeostasis can develop into a lethal *triad*, which involves hypothermia, acidosis, and coagulopathy, and death is imminent (50, 52).

## The First Incision

Besides cardiopulmonary bypass, the cutting, burning, fracturing and stretching of tissues induce an additional repair process, mediated by inflammatory responses and cell regeneration, which consumes energy and reserves. … The burden on some of them is substantial: a single sternotomy might reach the equivalence of a long bone fracture.

Prêtre (53)

### Local tissue trauma

**Figure 1:**
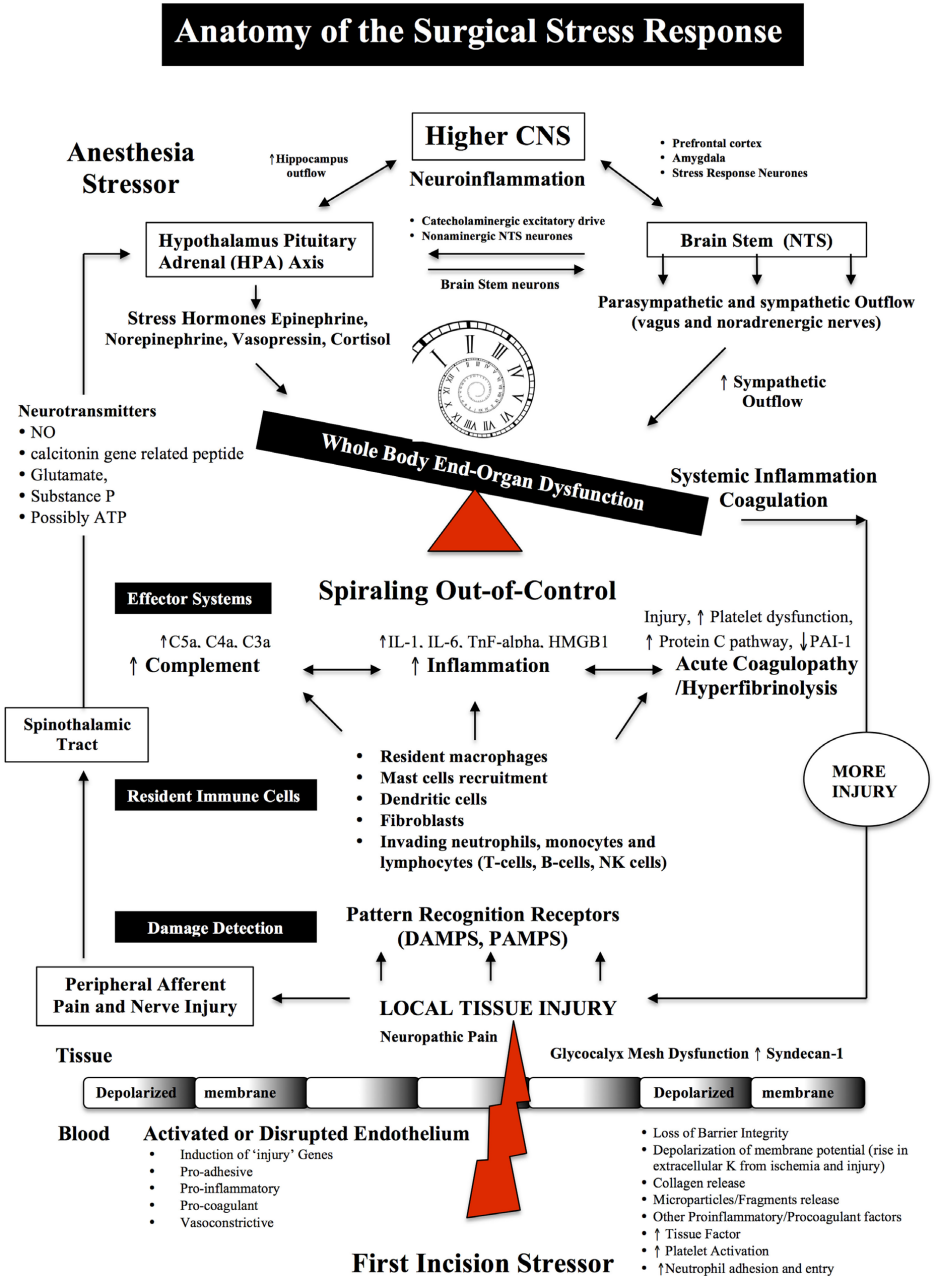
**Anatomy of the Surgical Stress Response**. Surgical stress triggers a wide and varied response at multiple levels depending on the type and duration of surgery, anesthesia and the patient’s age, gender and prior health status. The early drivers of the stress response are sterile local injury, afferent nerve cell firing, activation of the Hypothalamic-Pituitary-Adrenal (HPA) axis, Nucleus Tractus Solitarus (NTS), endothelial dysfunction and inflammation. Damage signals (also termed danger-associated molecular patterns or DAMPs and alarmins, e.g., heat shock proteins, adenosine, HMGB-1) are generated from tissue injury and detected by resident and non-resident immune cells. The key pro-inflammatory cytokines are IL-1, IL-6, and TnF-alpha and a complex interactions with complement. The primary goal of the acute immune response is wound healing and to prevent pathogen invasion. It is a restorative process that involves four phases: coagulation, inflammation, proliferation, and remodeling. Each phase of repair is predominately mediated by immune cells, cytokines, chemokines, transcription, and post-translational pathways (Tables 1 and 2). However, during major trauma, the early repair process can be overexpressed and lead to further injury, if not held in check. Peripheral nerve injury and pain induce afferent mediators and neurotransmitters to the spinal cord and central nervous system (CNS) and produce stress hormones, which exacerbate the stress response during major surgery.

**Figure 2:**
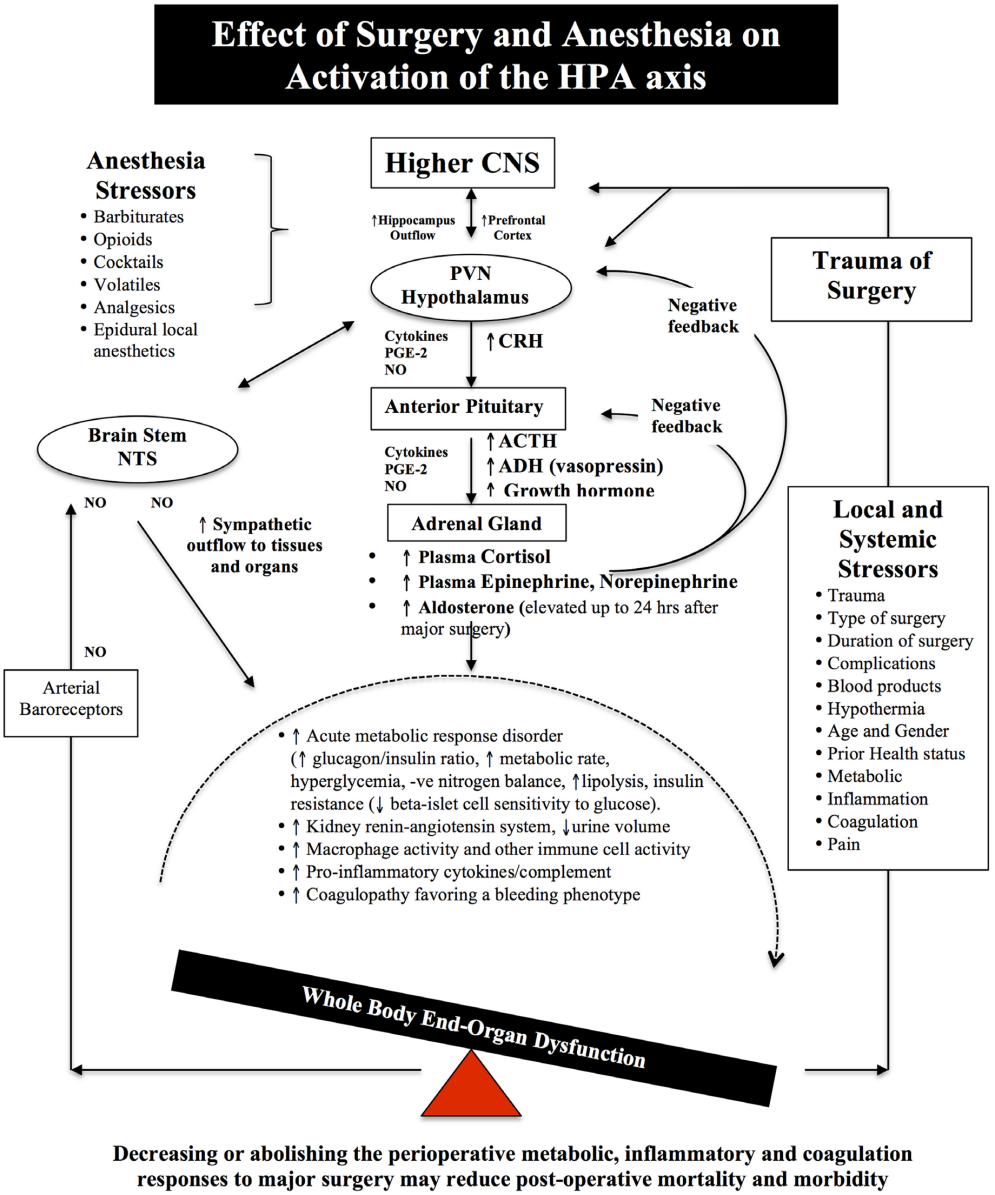
**Schematic of the HPA axis and the Stress Response to Surgery**. Different anesthetics have different effects on the HPA axis and immune system (see Effects of Anesthesia on the Surgical Stress Response). During surgical stress, activation of the HPA axis is controlled by a relatively small number of neurons located in the paraventricular nucleus (PVN) of the hypothalamus. These neurons release neural factors, such as corticotrophin-releasing hormone (CRH) and arginine vasopressin (AVP) into the hypophyseal portal circulation, which stimulates the anterior pituitary gland to release ACTH into the blood and activates the adrenal gland to release the stress hormones, catecholamines, and cortisol. Under normal exposure to stress, the HPA axis is held in check via multiple negative feedback mechanisms. However, during major surgery, trauma, infection or burns, imbalances occur and the action of stress hormones are potentiated by cytokines IL-1beta, IL-6 and TNF-alpha, prostaglandin-2 (PGE-2), and nitric oxide (NO), which predispose the body to further injury from ischemia, inflammation, and coagulopathy. Older surgical patients appear to be more vulnerable to surgical stress because their hypothalamus and pituitary are less sensitive to negative feedback from both cortisol and ACTH (7–9). The medullary Nucleus Tractus Solitarus (NTS) is also influenced by the stress response as it receives sensory neural inputs from the arterial baroreceptors, integrates this information with the hypothalamus, and other parts of the brain, and regulates the sympathetic and parasysmpathetic outflows to the body (7, 64–72).

The first surgical incision induces localized injury to tissues, afferent nerves, pain receptors and blood vessels (Figure 1). Nerve damage leads to afferent signals from the injury site to the brain and stimulation of the HPA axis (Figures 1 and 2). Local coagulation necrosis, microparticle release, endothelial damage, immune cell activation, localized cell ischemia, edema and metabolic dysfunction all contribute to a succession of rapidly cascading events from a local to a systemic phenomenon (54–60). Hypoxia also contributes to injury, in part, through the activation of hypoxia-inducible factor (HIF-1) and potentiation of NF-kappaB, a master regulator of genes involved in innate immunity, inflammation, and apoptosis (59, 61). The degree of trauma will be affected by the invasive nature of surgical procedure (56). Laparoscopic procedures, for example, have lower levels of trauma compared with open procedures, and lower surgical stress (62). Open surgeries include a median sternotomy, thoracotomy, laparotomy, abdominal hysterectomy and orthopedic surgery (54, 63). Cardiac surgeon René Prêtre equated the severity of a median sternotomy in cardiothoracic surgery with a long bone fracture (quote above).

### Activation of resident immune cells

At the incision site, resident and non-resident immune cells are the “first responders” to danger signals (Table 1) (60, 73–77). Resident immune cells are the macrophages, mast cells, dendritic cells, fibroblasts and lymphocytes (via lymph), and the non-resident blood-borne cells such as neutrophils and lymphocytes, which all produce a variety of cytokines, chemokines, proteases, leukotrienes, and nitric oxide (NO) in response to local injury (Tables 1 and 2) (75, 78–80). Mast cells are normally found in close association with blood vessels, lymphatic vessels, pain receptors and nerves, and on activation they degranulate with the release of inflammatory cytokines, histamine, and prostaglandins that initiate a neuropathic and nociceptive pain response and local changes in blood flow (60, 81–83). Circulating platelets also contribute to immune surveillance, damage control and amplification response to local injury (84–86). At the injury site, tissue factor (TF) activates platelets and coagulation pathways to reduce further blood loss. Normally, an effective inflammatory response isolates the area of injury, clears the injured cells/debris or kills invading pathogens and restores tissue function. However, the stress response can quickly become over-expressed and promotes further injury.

**Table 1:** **Immune cells involved in the detection, integration and healing following localized sterile injury**.

Type	Tissue location	Main functions
Macrophages	Resident	Macrophages are innate immune cells that ingest and process foreign materials, dead cells, and debris and recruit additional macrophages in response to inflammatory signals. Tissue-resident macrophages include brain (microglial), muscle, gut, kidney, alveolar, liver (Kupffer cells), bone (osteoclasts), and interstitial connective tissue (histiocytes) macrophages. Resident macrophages produce cytokines, chemokines, proteases, nitric oxide, and leukotrienes as part of their damage detection and amplification roles to local injury. Blunting the activation of resident macrophages/immune cells may reduce the stress response to surgery.
Mast cells	Resident	Mast cells are densely granulated effector cells of inflammation and immunity. They are located close to outer layers and barriers, such as epithelial borders, nerves, mucosal membranes, and vascular walls. At a nerve lesion, mast cells degranulate and release histamine, prostaglandins, leukotrienes, chemokines, and cytokines (TnF-alpha) to initiate the neuropathic and nociceptive pain response. Mast cells are involved in the local inflammatory response, vasodilatation, and plasma extravasation.
Dendritic cells	Resident	Like macrophages, dendritic cells are mononuclear phagocytes with multiple subpopulations that orchestrate host defense and wound healing to resolve local inflammation and support the resolution of fibrosis. An overexpression of inflammation causes increased recruitment of dendritic cells and collateral local and systemic injury.
Fibroblasts	Resident	Fibroblasts are involved in remodeling of extracellular matrix after injury. Excessive proliferation or secretion of extracellular matrix proteins may result from an overexpression of inflammation and pathologic progression of fibrosis and further tissue injury and adhesions.
Neutrophil	Blood-borne	Neutrophils migrate from postcapillary venules into the site of sterile tissue injury (or infection) and are the hallmarks of endothelial activation and acute inflammation. Their entry into the cell and release of cytokines, chemokines, proteases, and oxidants can contribute to further damage. Neutrophils can also promote fibroblast proliferation, aberrant collagen accumulation, and fibrosis. Inhibition of neutrophils and neutrophil-platelet interactions may be an important and effective target to reduce the local stress response during surgery.
Monocytes	Blood-borne	Increased monocyte reactivity occurs in trauma, infection, or burns. During local tissue damage monocytes are rapidly recruited to the tissue usually after neutrophils, where they differentiate into and replenish tissue macrophages or dendritic cells. Monocytes are equipped with a set of Toll-like receptors and possess unique scavenger receptors that recognize damage or pathogen-associated molecular patterns (DAMPS and PAMPS) and can clear apoptotic neutrophils by phagocytosis in a non-inflammatory process called efferocytosis.
Lymphocytes (B cells, natural killer cells and T cells)	Resident (lymph) and blood-borne	Damaged or necrotic cells alert the immune system to activate naïve T cells even in the absence of infective pathogens. T-cell induction, specifically CD4^+^ (Helper) and CD8^+^ (cytotoxic) cells, is now known to participate in recruiting neutrophils for the initiation of wound healing after sterile injury. Lymphocyte cells can also contribute to further inflammation and induce the up-regulation of endothelial cell adhesion molecules. The gut is a major source of lymphocytes and one of the “drivers” of major organ dysfunction and failure during the stress response to surgery.

**Table 2:** **Major cytokines, chemokines and danger signals involved in the acute stress response to sterile injury**.

IL-1	IL-1 has two subtypes, IL-1α and IL-1β, and they are key mediators of sterile inflammation. IL-1β is a potent pro-inflammatory cytokine produced mainly by tissue macrophages and can directly activate nociceptive fibers, and indirectly elicit the production of prostaglandins. IL-1β upregulates neutrophil- and monocyte-endothelial adhesion interactions. During acute stress, systemic IL-1β markedly increases brain IL-1β levels in the hippocampus, prefrontal cortex and hypothalamus. IL-1 also stimulates the hypothalamic-pituitary-adrenal (HPA) axis and alters the Nucleus Tractus Solitarus (NTS) sympathetic and parasympathetic outflow during stress.
IL-6	IL-6 is a sensitive, early marker of sterile tissue damage and acts as an inducer of the acute phase protein response. It also stimulates the HPA axis during stress and is a complex multifunctional cytokine that exerts pro- and anti-inflammatory effects.
IL-8	IL-8 is a chemokine produced by monocytes, T cells, neutrophils, natural killer cells, and somatic cells (e.g., endothelial cells, fibroblasts, and epithelial cells). It is inducible by IL-1 and TNF-alpha and recruits and activates neutrophils, promotes vascular smooth muscle cell proliferation and migration, and is involved in the chemotaxic and adhesion of monocytes to endothelial cells.
IL-10	IL-10 is an anti-inflammatory cytokine that serves as a brake to hyper-inflammation and immunosuppression by reducing the synthesis of proinflammatory mediators. Adenosine via A2A receptor-CAMP/PKA pathway inhibits IL-12 and TnF-alpha and stimulates production of IL-10 by antigen-presenting cells. Other protective cytokines include IL*-*17 that activates the differentiation of anti*-*inflammatory macrophages and phagocytosis of apoptotic neutrophils in response to IL-10 or glucocorticoids, and IL-21 and IL-22 play a role against tissue inflammation and protection. Induction of IL-10 expression, and the other anti-inflammatory cytokines, is a highly desirable therapeutic goal during major surgery.
TnF-alpha	TNF-alpha serves many functions in the inflammatory response with hormonal, metabolic, hemodynamic and neural effects. The cytokine is one of the early “danger” signals produced by resident macrophages, monocytes, dendritic cells, and neutrophils and T-lymphocytes. After Injury, Schwann cells produce TNF-alpha suggesting a role in neuropathic pain. TnF-alpha is also a chemotactic factor for fibroblasts and upregulates leukocyte-endothelial adhesion interactions, and is inhibited by adenosine. TNF-alpha may alter muscle metabolism by increasing amino acid availability, and is involved in insulin resistance. It also stimulates the HPA axis during stress and has anti-inflammatory properties.
NF-κB	NF-κB is an archetypal pro-inflammatory *pathway* activated by IL-1 and TnF-alpha. Once activated, the pathway induces proinflammatory genes, cytokines, chemokines, and endothelial adhesion molecules. NF-κB was once considered the “holy grail” as a target for new anti-inflammatory drugs, but it is now known to have anti-inflammatory properties. Thus NF-KB transcription factors regulate inflammation and orchestrate the immune response during sterile injury or following infection.
HMGB1	High mobility group box-1 is a ubiquitous nuclear protein loosely bound to chromatin and is released from macrophages and monocytes exposed to inflammatory cytokines. It also acts as a danger signal (DAMP) from sterile injury via loss of membrane integrity of damaged or necrotic cells and is an initiator of innate immunity. HMGB1’s surface receptor, RAGE (receptor for advanced glycation end products) promotes NF-κB activation, which is responsible for most events elicited by necrotic cells. HMGB1 may also be involved in restorative effects leading to tissue repair and regeneration. Active HMGB1 secretion also appears to be under autonomic nervous control with splenic macrophages being an abundant plasma source during the stress response. After major surgery, high plasma levels have been linked to cognitive decline.
Mitochondria	Mitochondrial damage is a rich source of danger signals (DAMPS), including mitochondrial DNA, formyl peptides, cytochrome C, and ATP. These micro-particles are potent stimulators of acute inflammation. Tissue ischemia and reperfusion can also activate complement by exposing mitochondrial proteins and cell phospholipids that are recognized by natural and locally occurring antibodies and autoantibodies.

### Inflammatory cytokines: IL-1, IL-6, and TnF-alpha

The main pro-inflammatory cytokines are IL-1, IL-6, and TnF-alpha (Table 2) (7, 76, 80, 87–91). IL-1 beta can directly activate nociceptive fibers within 60 s, and indirectly elicit the production of prostaglandins in response to injury (60, 78, 81, 88, 92). In addition to local effects, plasma IL-1, IL-6, and TnF-alpha stimulate the HPA axis leading to the release of cortisol and catecholamines and systematization of the stress response (68, 93). Interestingly, plasma IL-6 levels appear to correlate with the severity of surgical injury and post-operative complications (94). In 2006, Ishibashi and colleagues showed that IL-6 levels correlated with the length of a surgical incision (1.0 cm vs. 3.0 cm) with the larger incision leading to doubling of IL-6 values at 3 and 6 h (95). During colorectal surgery, plasma IL-6 and P-selectin were also shown to increase during surgery and peaked at day one after surgery (96). IL-6 also appears to play a dual role by providing a later “immunological brake” to restore homeostatic balance by up-regulating anti-inflammatory mediators and cytokine inhibitors, such as prostaglandin PGI_2_, IL-1 receptor antagonist (IL-1ra), IL-10, and transforming growth factor (TGF-b) (40, 77, 97). Thus, resolution of the local and systemic inflammatory responses is an emerging area of increasing complexity and involves immune cells, cytokines, the endothelium, and a multitude of negative feedback new mediators such as the resolvins, lipoxins, maresins, and NF-kappa beta pathways (40).

TNF-alpha is another major cytokine and participates in a diverse number of cellular and molecular events such as neutrophil-endothelial adhesive interactions, vasodilatation, microvascular leakage, edema, and oxidative stress (Table 2) (98–100). TnF-alpha, and other cytokines, activate the hepatic acute phase protein response with the release of C-reactive protein (CRP), pro-calcitonin, and C-3 complement factor (70, 92). Complement activation is an integral part of the stress response to surgical injury and intersects and amplifies the coagulation activation pathways (see Complement Injury Cascade) (84). Like IL-6, TNF-alpha serves a dual role in resolution of the inflammatory response via signaling through the TNF-R2 (p75) receptor (101). The timing of these pro- and anti-inflammatory roles during surgical stress and recovery are not well understood.

### Local coagulation and systemic coagulopathy

Local clotting, as mentioned, is initiated by exposure of platelets to the subendothelial components and TF activation of plasma factors VII/VIIa via the extrinsic pathway, which in turn leads to the generation of thrombin, fibrin deposition, and clot formation (102–104). Other proteins, such as von Willebrand factor, facilitate the binding of platelets to the injured vessel wall. TF is expressed by the injured blood vessel wall (adventitial fibroblasts, smooth muscle cells and pericytes), platelets, activated endothelium, macrophages, neutrophils, circulating microparticles, and epithelial cells (105, 106). Importantly, TF is essential for hemostasis, but *uncontrolled* expression of TF can lead to coagulopathy (107). A second clotting pathway, known as the *Intrinsic Pathway*, appears to be more important in the growth, amplification and elongation of clot formation, rather than the TF-activated initiation (108). The intrinsic pathway involves clotting factors within the blood itself combined with cell fragments, microparticles or damage-associated molecular pattern molecules (109). The relationships between the extrinsic and intrinsic pathways, and their different timings and systemic manifestation during major surgery are not well understood.

In major surgery, a common complication is hyperfibrinolysis (110). According to the STS database, up to 50% of cardiac surgery patients require blood product transfusions (111), and 5–7% will lose in excess of 2 l in the first day postoperatively (112, 113). About 50% of blood loss is due to identifiable surgical bleeding, and the other 50% is due to a hypocoagulopathy associated with surgical trauma, heparinization, and CPB (112, 113). Surgical hypothermia further exacerbates hemorrhagic, cardiovascular, and infectious complications compared to normothermia (114). In non-cardiac surgery, coagulopathic perioperative bleeding is also a major problem. In a recent prospective, multicenter observational cohort study of 1134 consecutive patients with coronary stents, 9.5% experienced postoperative hemorrhagic complications after non-cardiac surgery, and 12% of these died (115). In general, trauma-induced postoperative coagulopathy is associated with extended hospital stays and three times higher healthcare costs (116). The impact of the first incision on triggering the local inflammatory and coagulation responses appears to be overlooked, as there is surprisingly little information on the nature and timing of these events, and when they become systemically expressed. New pharmacological therapies for prevention or early correction of inflammation and coagulopathy are urgently sought.

### Complement injury cascade

Complement activation is another integral part of the stress response to surgical injury (Figure 1). The blood complement system comprises a group of at least 30 soluble plasma and membrane bound proteins and can be activated in two main ways; (1) as a component of the innate immune cascade in response to aseptic tissue injury (i.e., antibodies or T cell receptors not involved), and (2) from the adaptive immune response when antibodies (IgG or IgM) binds to antigen at the surface of a cell (117–121). Along with the local inflammatory and coagulation responses to injury, the complement system is part of an ancient innate immune response to trauma, ischemia, hemorrhage, burns, infection, and autoimmunity (120, 121).

Complement proteins are synthesized in the liver and by extra-hepatic tissue macrophages, blood monocytes, and epithelial cells. Local production and activation of complement makes a significant contribution to local tissue inflammatory injury (necrosis), coagulopathy, and augmentation of the adaptive immune response (117, 122). The two main mechanisms of complement cell damage include membrane attack complex (MAC) and cell bound ligands C4b and C3b that activate innate immune cells such as neutrophils bearing complement receptor (117), and second, from small activation fragments known as anaphylatoxins (C3 and C5 convertases) that further promote the influx and activation of neutrophils, cytokines, chemokines, adhesion molecules, and cascade mechanisms (117). Tissue ischemia and reperfusion can also activate complement by exposing cell phospholipids and mitochondrial proteins that are recognized by natural and locally occurring antibodies and autoantibodies (118, 120, 122, 123). CRP, an acute phase pro-inflammatory mediator and activator of the classical complement pathway, has been shown to increase up to 1000-fold in human plasma following tissue injury (120, 124).

In cardiac surgery, the timing of complement system activation was thought to occur only when the patient’s blood was in contact with pro-inflammatory plastic tubing of the CPB circuit (125, 126). However, Gu and colleagues found that plasma complement and inflammation occurred soon after the first chest incision (127). This was further supported by their observation that complement was activated in those patients who *did not receive* CPB but related to the first incision (127). In addition, a smaller anterolateral thoracotomy was associated with reduced complement activation, and lower IL-6, compared to the median sternotomy. Interestingly, IL-6 was not elevated in plasma until the end of the operation, and Gu’s group suggested that the “incision” trigger was a tissue type plasminogen activator, which is known to stimulate complement (127). Unfortunately, the study did not measure plasma IL-1, TnF-alpha, or other markers of inflammation and coagulation at baseline and before or after CBP. As with other components of the innate immune response, excessive activation of complement pathways damages healthy tissues from “friendly fire,” and exacerbates the surgical insult (121). Further research is required to better understand the mechanisms of hyper-expression, the timing of systemic organ damage and failure, and to develop novel therapeutic strategies, such as complement inhibitors, to possibly improve surgical outcomes (126).

### Role of histamine, nerve growth factors and local opioids

Histamine is locally released from mast cell granules, and nerve growth factor is released from damaged nerves, which activate peripheral nerves that either terminate in the brain or spinal cord dorsal horn resulting in pain facilitation (128). Histamine release also appears to be directly related to changes in the cardiovascular system that are often seen during anesthesia (129). Simultaneously, endogenous analgesic mechanisms are activated including anti-inflammatory cytokines, endocannabinoids, and opioid peptides. Opioid peptides such as endorphins, enkephalins, and dynorphins are produced by immune cells such as leukocytes and can be released locally in the inflamed tissue on stimulation with IL-1 or from corticotropin releasing factor that drives the body’s response to stress (130). Following release, opioid peptides bind to receptors on peripheral sensory neurons and produce analgesia in animal models and humans (131).

## Effects of Anesthesia on the Surgical Stress Response

It is critical to recognize that certain stressors may act on the brain even in the unconscious state - this is true of the anesthetic itself and the hormonal, metabolic and inflammatory mediators of the surgical stress response.

Borsook et al. (132) p. 607

### Blunting the stress hormones

Notwithstanding the difficulty in separating the stress effects of anesthesia from the surgery itself, there is a general consensus that most anesthetics reduce the neuroendocrine response. The degree of reduction is difficult to assess and most likely depends upon the anesthetic’s mode of action, dose and duration of use (Figure 2). As mentioned in Section “Cuthbertson’s “Ebb and Flow” Injury Hypothesis and the HPA axis,” the central integrative “hub” controlling the stress response is the HPA axis that controls catecholamine and cortisol production, and the Nucleus Tractus Solitarus (NTS) that control sympathetic-parasympathetic outflows (7, 64–72). In addition, prostaglandin and NO production in higher centers, such as the cortex, hippocampus, and amygdala and periphery, are all involved in the regulation of HPA axis and NTS under basal and stress conditions (71) (Figures 1 and 2).

Blunting the stress response includes a reduction in the production of the following major hormones or autocoids as possible clinical endpoints:

- *pituitary hormones*: adrenocorticotropic hormone (ACTH), growth hormone (GH) and antidiuretic vasopressin, beta-endorphins;
- *adrenal catabolic hormones*: cortisol and catecholamines;
- *pancreatic hormone*: glucagon;
- *prostaglandins* (e.g., PGE-2).

The most common general anesthetics include the barbiturates (thiopental), opioids (fentanyl, remifentanil, sufentanil), benzodiazepines (midazolam), dissociative anesthetic agents (ketamine) and intravenous propfofol, etomidate, and clonidine (7, 68). No sedation or drug cocktail, however, offers complete “stress-free anesthesia” (133). Opioids appear to be the most powerful suppressors of HPA, and particularly short-acting etomidate, which suppresses corticosteroid production, cortisol release, catecholamines, and aldosterone release, which may last between 8 and 22 h after surgery (68, 134). Propofol-remifentanil cocktail also blunts the stress response but to a lesser degree (135). Despite their individual effects, many of the current anesthetic combinations, particularly used in cardiac surgery, still lead to persistent elevations of 2– 6 fold in cortisol, GH, and norepinephrine lasting around 2 days (136). The volatile anesthetics halothane, isoflurane, sevoflurane, and nitrous oxide have been reported to be less effective in blunting the stress response (137, 138). However, sevoflurane appears to be more effective at blunting the inflammatory response than isoflurane (139). Other anesthetics, such as thiopental, ketamine, and opioids, possess some anti-inflammatory and anti-oxidant properties (140), however, prospective, randomized clinical trials are required to examine if these differences are clinically significant (138, 141).

### Optimizing anesthesia and analgesia to reduce pain and the stress response

Nearly 90% of surgical patients claim to experience moderate-to-severe postoperative pain after major surgery (142). Neuropathic and nociceptive pain contributes to and amplifies the stress response by increasing inflammation, coagulation disorders, organ hypoperfusion, decreasing wound healing, and possibly cognitive dysfunction (77, 143, 144). To address this problem, the concept of pre-emptive analgesia has been introduced in recent years (142, 145, 146), which combines general anesthesia with epidural and/or intravenous (IV) infusion agents (e.g., opioids, local anesthetics). This analgesic strategy also reduces the need for steroids, which have been associated with increased infections (147, 148), and opioid use, which reduces GI post-operative complications (149). The recent emphasis on pre-emptive analgesia for procedure-specific pain management via CNS and peripheral desensitization appears to be improving post-operative care (150), and similar to George Crile’s *anoci-association* and “stress-free surgery” proposed over 100 years ago (see George Crile and “Stress-Free” Surgery).

### Stress-induced diabetes

Stress hormones from the HPA axis and increased sympathetic outflows from the NTS also lead to hyperglycemia and insulin resistance (Figure 2), which may persist for several days from higher levels of cortisol, catecholamines, GH, and pro-inflammatory cytokines (66, 72, 151). This condition has been termed the “diabetes of injury” (5, 6) or “critical illness diabetes,” and is common after severe surgical trauma, multiple injury trauma, burns, or infection (5). Surgical stress leads to net glucose production, a decrease in uptake and tissue utilization and/or a decrease in pancreatic β-cell responsiveness to insulin signaling (68, 152, 153). It is also associated with endothelial activation, coagulopathy, cardiac dysfunction, arrhythmias, immunosuppression, and slower wound healing times (72, 154). In addition, those patients who are already diabetic and undergo cardiac surgery, have a 24% higher risk of readmission for cardiac-related issues, deep sternal wound infections and post-operative strokes, and a 44% higher risk for rehospitalization for any cause (155).

## Cognitive Dysfunction

The pathogenesis of postoperative cognitive dysfunction (POCD) is multifactorial and future studies should focus on evaluating the role of postoperative sleep disturbances, inflammatory stress responses, pain and environmental factors.

Krenk et al. (156), p. 951

### A persistent complication of major surgery

Cognitive dysfunction remains a continuing complication in the aged and very young. In patients 60 years or older undergoing cardiac surgery, cognitive dysfunction occurs in 30–52% of cases (157), and may last up to 5 years (158, 159). In non-cardiac surgery patients, Price and colleagues reported cognitive dysfunction in 56% of cases, and 25% in older patients after 3 months (160). A number of systematic reviews generally support these findings but cautioned that differences may also reflect differences in the neuropsychological tests to assess cognitive dysfunction (161). In pediatric patients after cardiac surgery, 5–10% acquire some form of cerebral dysfunction (162), although it usually resolves faster than in older patients (156). In addition, major surgery in very low-birth-weight infants is independently associated with a greater than 50% increased risk of death or neurodevelopmental impairment and anesthetics are believed to be involved (163).

**Figure 3:**
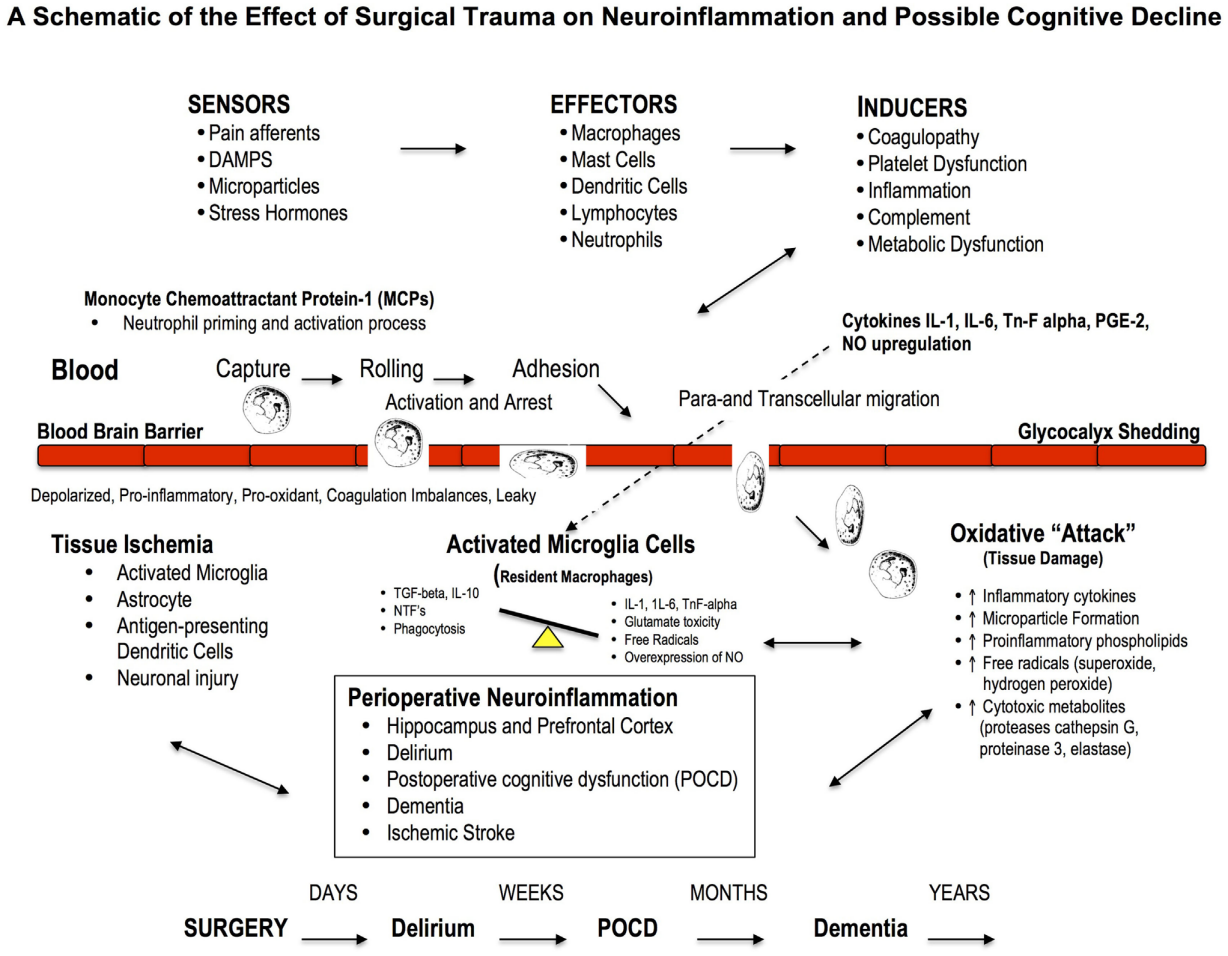
**Effect of Major Surgery on the Blood Brain Barrier and Neuroinflammation**. The blood brain barrier (BBB) is the body’s natural “firewall” to protect against unwanted incoming agents entering the CNS from the general circulation. During major stress and trauma, the BBB is particularly vulnerable to attack from inflammatory cells and cytokines (156). A breach can lead to neuroinflammation and activation of microglial cells, the brain’s resident macrophages, which may lead to further injury and cognitive dysfunction. The three most common types of cognitive dysfunction are delirium, postoperative cognitive dysfunction (POCD) and dementia (168). Delirium is normally defined as a transient loss of mental attention and orientation in the hours after surgery; dementia is a series of syndromes associated with global deterioration of cognitive ability lasting months to years; and POCD is the deterioration in performance (156, 169). POCD is a more subtle condition and longer lasting than delirium and can include post-traumatic stress disorder after an ICU stay (156). Stroke is another leading cause of severe, long-term cognitive disability after major surgery and occurs in around 2% of patients (167, 170), and 20% of these occur within the first two postoperative days (171).

The factors responsible for cognitive dysfunction are complex and include transient hypoperfusion, hypoxia, ischemia-reperfusion injury, low preoperative hemoglobin levels, fluid overload, blood transfusions, microemboli, perioperative pain, hyperglycemia, and large swings in CNS temperature (77, 157, 164–167). However, the underlying factor believed to be responsible for cognitive dysfunction underpinning all these appears to be inflammation (Figure 3).

### Neuroinflammation: A driver of cognitive loss

Neuroinflammation is associated with local or widespread activation of microglia, which are the resident *macrophages* in the brain and spinal cord, and early responders to injury (77, 86, 172, 173). A large part of early cognitive decline appears to occur within the hippocampal and prefrontal cortex areas (174, 175), and may involve: (1) the acute activation of the phagocyte NADPH oxidase (PHOX) found in microglia, (2) expression of the inducible nitric oxide synthase (iNOS) in glia, and (3) microglial phagocytosis of neurons (176). In addition, an acute overexpression and secretion of brain-derived neurotrophic factor (BDNF) by microglial cells, and their intraneuronal pathways, have been implicated as potential mediators of inflammation and hippocampal neuronal dysfunction (e.g., decreased neurogenesis, synaptic plasticity and long-term potentiation of stable memory formation) (174, 175, 177). Recently, Hovens further showed microglia activation in the hippocampus and prefrontal cortex persisted for one week after major surgery (175).

Neuroinflammation and cognitive loss may also occur from damage to the blood brain barrier (BBB), which forms the nexus between the central and peripheral nervous and circulatory systems. A breach to the BBB may facilitate entry of blood-borne immune cells (e.g., neutrophils, lymphocytes) and cytokines (e.g., IL-1, IL-6, or TnF-alpha) and prostaglandins (PGE-2), which all exacerbate inflammation (166, 178) (Figure 3). Based on cerebral MRI, BBB disruption and leakiness have been reported in around 50% of cardiac surgery patients with CPB (179, 180). Excessive leakage of fluid and proteins is also a common occurrence following traumatic and ischemic brain injury, and is more pronounced in the elderly (181). The presence of cerebrospecific protein S100β in serum is an important indicator of cerebral damage (182). Platelets also accumulate in the CNS parenchyma and release pro-inflammatory factors, and play a role in the pathogenesis of cognitive decline (86). In 2012, He and colleagues found in aged rats after a splenectomy that the BBB was damaged, and the hippocampus had high levels of upregulated HMGB1 and the receptor for advanced glycation end products (RAGE) compared to controls (183). Since control rats received the same general anesthesia, the cognitive dysfunction was attributed to the surgery. Serum HMGB1 and IL-6 levels increase significantly after major gastrointestinal (GI) surgery in elderly patients and have been associated with cognitive decline after surgery (184). Unfortunately serum S100β was not measured. Currently there is no adequate protective therapy for cognitive decline.

## Effects of Major Surgery on the Other Organs

### Myocardial injury

Among adults undergoing non-cardiac surgery, myocardial injury after non-cardiac surgery is common and associated with substantial mortality.

Botto and Alonso-Coello (185), p. 564

Globally each day over 700,000 adult patients undergo non-­cardiac surgery, and around 30% will have some form of pre-existing coronary artery disease (14). In this pre-existing coronary artery disease group, 3.9% or over 8000 patients per day will carry the risk of suffering a major perioperative cardiac event (186). The most common event is a myocardial infarction (MI), which is associated with an in-hospital mortality of 15–25% (186, 187). In a recent international prospective cohort study of over 15,000 non-cardiac surgery patients, 8% of patients over 45 years suffered myocardial injury, based on elevated blood troponin T levels, and 10% of these (120 patients) died from a cardiac event within 30 days (185).

Myocardial ischemia can also result from excessive catecholamine production, alterations in baroreceptor set-point receptivity, and reduced heart rate variability, especially in older patients (188). Plasma catecholamines are associated with higher cardiac troponin levels, and some perioperative thrombotic states can lead to coronary plaque disruption and MI (187). Recent trials using low-dose presynaptic alpha2-adrenergic agonist, clonidine, designed to blunt norepinephrine production, failed to reduce the incidence of perioperative MI (189). However, pretreatment with cyclosporine-A in patients undergoing elective CABG surgery did reduce the perioperative myocardial injury during longer CPB operations (190). Further drug discovery to reduce perioperative “adrenergic stress” and protect the heart from myocardial injury is urgently needed (185).

Another adverse event of major surgery is low CO syndrome, particularly following pediatric and adult cardiac surgery (191). Cardiac depression occurs in many other acute critical states, such as hemorrhagic shock, infection, sepsis, or burns (192–194). While the mechanisms are not fully understood, a cardiac inflammatory response and altered myocardial Ca^2+^ handling are thought to be involved (191). Interestingly, in animal models, infusion of pro-inflammatory cytokine TNF-alpha has been shown to induce myocardial depression and ischemic-reperfusion injury (195, 196). Similarly, during major surgery it is proposed that the gut may be a possible source of high levels of TnF-alpha because in acute injury models, the gut and mesenteric lymphatics are major contributors to systemic inflammation and multiple organ dysfunction (39, 192, 197, 198). In addition, rat studies show that ligating the mesenteric duct has improved CO during acute burn trauma, further suggesting a “gut-derived factor” may be responsible for low CO syndrome (192, 193). The heart itself may contribute to depressed function because TnF-alpha can also be produced by resident macrophages (199). Other cardiovascular complications during major surgery include arrhythmias, unstable angina, cardiac arrest, heart failure, hypertension, and stroke (200).

### Renal dysfunction

Postoperative acute deterioration in renal function, producing oliguria and/or increase in serum creatinine, is one of the most serious complication in surgical patients.

Brienza et al. (201), p. 2079

Cardiac dysfunction can negatively impact on every organ and tissue of the body. The kidneys normally receive 20% of the CO and insufficient flow can lead to an abrupt reduction in glomerular filtration rate and acute kidney injury (AKI) (202). AKI occurs in 1% of non-cardiac surgical patients, and up to 30% in cardiac surgery patients and around 3% of these patients may require dialysis (203, 204). Patients with AKI requiring renal replacement therapies have mortality rates in excess of 40–50% (202). This condition is strongly associated with ischemia, systemic inflammation, emboli, GI bleeding, respiratory infections, and sepsis (205). To date, no single perioperative strategy has demonstrated a therapeutic benefit to improve CO and prevent renal injury after CPB surgery (202, 203).

### Perioperative pulmonary injury

Perioperative pulmonary complications may equal or outnumber cardiac events.

Johnson and Kaplan (206)

An unappreciated fact is that pulmonary perioperative complications may equal or outnumber cardiac complications (206–208). Postoperative lung dysfunction occurs in 3–10% of patients after elective abdominal surgery (209), 2–7% after thoracic surgery (210), and 30–50% in patients after cardiac surgery (211). Anesthesia induction itself can lead to ventilation/perfusion mismatch, atelectasis and impaired oxygenation (208, 209). *Atelectasis occurs in around 90% of all major surgeries and can persist for several days and predispose the patient to pulmonary infection* (212). Abdominal or thoracic surgery is often associated with 20% or more loss of functional residual capacity from diaphragmatic dysfunction, decreased chest wall compliance, and pain-limited inspiration, which may not resolve itself for a week postoperatively (212).

A devastating complication of cardiac surgery is acute respiratory distress syndrome (ARDS), which occurs in 2–3% of low to medium risk patients, and up to 20% in high-risk patients (213, 214). Post-operative ARDS carries a mortality of 40–80% (214). A milder form of ARDS is acute lung injury (ALI) and it generally appears within 2 days of surgery (137, 215). Both ARDS and ALI are part of a systemic disorder associated with microvascular endothelial permeability dysfunction, inflammation, and widespread organ involvement including the heart and cytokines from the GI tract (216, 217). A recent study involving 1817 patients found that transfusion-related ALI, termed TRALI, occurred in 1.4% of patients undergoing major surgery with higher incidences reported (2–3%) after vascular and transplant surgery (218). In addition, the same group found that transfusion-associated circulatory overload (TACO) was a leading cause of transfusion-related fatalities with an overall incidence of 5.5% and highest in vascular (12.1%), transplant (8.8%), and thoracic surgeries (7.2%) (219).

Pulmonary complications after CPB have been known since the mid-1950s because the lungs are almost entirely excluded from the systemic circulation, and alveolar blood is nearly “static” other than receiving residual blood flow from the bronchial arteries (137). During long cross-clamp times, the lungs are under enormous ischemic and inflammatory stress, which is further exacerbated when the cross-clamp is released and the heart and lungs are “reperfused” with oxygenated blood. Re-oxygenation, mitochondrial free oxygen radicals and increased “gut” cytokines further exacerbate the inflammatory response. Neutrophils and macrophage infiltration damage Type I cells leading to alveolar flooding with protein-rich fluid (edema) and Type II cells leading to reduced surfactant production, both of which predispose the lung to ALI and ARDS (214). Recent clinical trials with β2 agonists to increase alveolar fluid clearance and “immunonutrition” with omega-3 fatty acids have been disappointing (215). Novel therapies are required to protect the lung from injury during major surgery, and in particular CPB surgery, and the possibility of using mesenchymal stem cells to form new Type 1 and 2 cells are being investigated.

### Gastrointestinal injury and the “cytokine storm”

We believe that there is currently no unifying hypothesis that encompasses the diverse ways in which the gut influences outcome in critical illness.

Clark and Coopersmith (198), p. 385

During major surgery, excessive sympathetic activation, and inflammatory and coagulation imbalances can cause gut dysfunction (220). The GI tract is a powerful immunologically active organ, and plays a key role in maintaining the health of the brain, heart, and lung under normal conditions (38). GI dysfunction ranges from mild complications such as ileus to less common but severe hypoperfusion and ischemic complications, which all carry a high mortality of 60–80% (221). Like the kidneys, the GI tract receives about 20–25% of the resting CO (38). However, during surgical stress sympathetic alpha-adrenergic stimulation can constrict the mesenteric artery and intestinal and intrahepatic portal veins leading to hypoperfusion of the gut and associated organs (liver, pancreas, and spleen), and this response may be potentiated by the posterior pituitary hormone vasopressin (38, 222). During CPB, the initial rise in circulating catecholamines can decrease hepatic perfusion by 20–45% and splanchnic blood flow by approximately 20% (38, 222). These falls in blood perfusion can lead to ischemic complications especially during longer more complex operations. GI injury is an important component of the SIRS and can lead to sepsis, MOF, and death.

The gut is so critically important to the health of the surgical patient that Meakins and Marshall described it as the “motor” of MOF (198) p384. This “motor” can magnify the systemic inflammatory response from: (1) a breach in intestinal epithelium permeablity, (2) activation of the cytokine-mediated GI immune system, and (3) bacterial and endotoxin translocation from the lumen into the peritoneum via the portal circulation (198, 223). A breach can have such a profound effect because in a normal healthy intestine there are more than a hundred trillion (~10^15^) bacteria, viruses, and fungi, which outnumbers the cells in the human body by tenfold (224, 225). In addition, there are more lymphocytes associated with the intestine than there are in the rest of the human body. Commensal bacteria at the intestinal epithelial interface are believed to regulate the level of NF-κB activity and thereby affect the GI mucosal immune balance (226). During major surgery, and other forms of trauma, if this balance is perturbed complications can arise from a “cytokine storm.” As the “storm” develops more circulating neutrophils are attracted to the interstitial compartments and damaging O_2_ free radicals, proteolytic enzymes and more cytokines are produced, tight junctions are breached, mucosal cells proceed to necrosis and apoptosis and endotoxemia and sepsis can develop.

The cytokine “storm” from the gut may also be responsible for perioperative cardiac, lung, brain, and other organ dysfunction. As mentioned, cardiac depression, ALI and ARDS are related to an overexpression of the acute immune response. Following major GI surgery, Takahata and colleagues correlated the duration of SIRS and pulmonary dysfunction with the appearance of serum cytokine HMGB-1 levels (227). Other studies implicate intestinal phospholipase A2 generated arachidonic acid and its subsequent 5-lipoxygenase products to pulmonary injury (216), while others suggest an alteration of the T-helper 1/T-helper 2 cytokine lymphocyte balance (228). It is also becoming apparent that CNS imbalances leading to low heart rate variability (229, 230) are associated with increases in systemic inflammation, and possibly involve TNF-alpha production by spleen macrophages (231). Interestingly, increasing parasympathethic outflow via vagal cholinergic stimulation appears to reduce inflammation in ileus following intestinal manipulation in animal models (229, 230). Other factors that modulate the gut may come into play and include vasoactive intestinal peptide, glutamate, and NO, all of which have been shown to modulate immune cells (230). Maintaining a healthy GI tract is imperative to bolstering a patient’s defense against the stress of major surgery.

### Liver injury during major surgery

The clinical task to minimize perioperative hepatic cellular injury is challenging for anaesthetists and intensivists alike.

Beck et al. (232), p. 1070

Mild liver dysfunction in patients without liver disease is common following major surgery (232, 233). Acute “ischemic hepatitis,” as it is termed, is a diffuse injury that arises secondary to hypoperfusion (hemodynamic instability) and hypoxia, and is exacerbated by systemic inflammation. Typically, this condition resolves within a few days. More serious complications can arise in patients with preexisting liver disease and cardiac dysfunction. The liver is particularly vulnerable to low flow because of its high O_2_ requirement and complex portal vein and hepatic artery network (38, 232, 234). The portal system from the stomach, spleen, pancreas, intestines, and omentum supplies 70–80% of blood to the liver at very low pressures (5–10 mmHg) (234), and patients with chronic hepatic congestion or cirrhosis are vulnerable to hypoxic liver injury (232). Other risk factors include the use of CPB, total time on bypass, non-pulsatile flows, fluid overload, and the type of anesthetic and perioperative vasopressor support (233). As mentioned above (see Gastrointestinal Injury and the “Cytokine Storm”), CPB can lead to decreases in splanchnic blood flow by ~20% and hepatic arterial blood flow by up to 45% (222), which may lower venous return and therefore CO resulting in systemic ischemia (38, 235). Anesthetic agents can also reduce hepatic artery blood flow by 50–70% (232, 233). Agents such as isoflurane, desflurane, sevoflurane, and propofol are preferred in patients with liver disease because they have less impact to reduce blood flow compared to other inhaled anesthetic agents (232, 233).

Systemic inflammation is perhaps the most common underlying factor leading to acute and chronic liver dysfunction (236). The patient with liver disease is already in a pro-inflammatory state of “rebalanced hemostasis” that can lead either to excessive bleeding or thrombotic complications (237). Like the GI tract, the liver plays a critical role in immune defense against surgical stress (238, 239), and is involved in maintaining adequate venous return and CO (38, 235). New therapies are urgently required to protect the liver and maintain adequate blood flow through the splanchnic system to support cardiac function during major surgery.

### Immunosuppression and susceptibility to infection

General anesthesia accompanied by surgical stress is considered to suppress immunity, presumably by directly affecting the immune system or activating the hypothalamic-pituitary-adrenal axis, and the sympathetic nervous system.

Kurosawa and Kato (240)

Immunosuppression is common following major surgery secondary to systemic inflammation and coagulopathy (87, 97). A dramatic depression of cell-mediated immunity predisposes the patient to slow healing, multiple organ injury, infection, and sepsis (58, 241, 242). In cancer patients, emboli dispersal from surgery along with post-operative immunosuppression can lead to further tumor metastases (243). Recent studies implicate impaired natural killer (NK) cell response, lymphocyte depression, and monocyte deactivation as playing major roles in mediating immunosuppression after major surgery (57, 244). Trauma appears to increase the expression of T-helper 2 (Th2)-stimulated lymphocytes and their cytokines resulting in a lower plasma Th1/Th2 cytokine ratio, which is believed to be associated with immune paralysis (228, 239).

A lower plasma Th1/Th2 cytokine ratio is also mediated by the stress hormones glucocorticoids and norepinephrine via activation of the HPA axis and mast cell-histamine reactions (239, 245, 246), and possibly from increased NTS sympathetic outflows. Immunosuppression may be exacerbated by persistent perioperative splanchnic and liver hypoperfusion and gut cytokine production (247–249). Suppressed cellular immunity can continue for 3–10 days post-operatively in patients who have undergone major surgery, but not minor surgery (57). As noted in the Section “Inflammatory Cytokines IL-1, IL-6, and TnF alpha” and Table 2, post-operative immune-competence can be routinely evaluated by measuring plasma levels of interleukins (1β, 2, 6, 8, 10, 12), TNF-α, stress hormones, CRP, and the T-lymphocyte profile (62).

## Current Perioperative Therapies: The Good, Bad and the Ugly

Cardiologists frequently advise on perioperative care for non-cardiac surgery and require guidance based on randomized controlled trials that are not discredited by misconduct or misreporting.

Nowbar et al. (250), p. 138

The goal of perioperative therapies is to reduce or prevent surgical “stressors” from developing and to accelerate recovery (251–253). Three major therapies that have attracted a lot of clinical interest to improve perioperative protection are: (1) statins, (2) beta-adrenergic blockers, and (3) calcium-channel blockers.

### Statins

Statins are hydroxymethylglutaryl (HMG)-CoA reductase inhibitors and powerful cholesterol-lowering agents (253). Perioperative interest comes from their pleiotropic ability to potentially decrease oxidative stress, inflammation and thrombosis via inhibition of G proteins and induction of transcription factors (251, 254). The first line of evidence supporting statin therapy originated from a landmark, non-surgical Heart Protection Study involving over 20,000 high-risk patients with coronary artery disease or diabetes. In those patients who received 40 mg simvastatin daily there was a significant reduction in all-cause mortality (12.9 vs. 14.7%) from MI, stroke, and the need for coronary and non-coronary revascularization (255, 256). However, these data were challenged in a meta-regression analysis of Robinson and colleagues who compared non-statin and statin trials between 1966 to October 2004 and concluded statins do not appear to contribute a cardiovascular benefit beyond their well proven lipid lowering abilities (257). More recently, in high-risk patients undergoing non-cardiac surgery, de Waal and colleagues concluded there is insufficient data to support final recommendations on perioperative statin therapy (258).

Despite the controversy, some groups argue that statins are underutilized during major surgery (253). For example, Paraskevas and colleagues concluded from Medline searches that statins reduce the incidence of postoperative and postprocedural renal insufficiency and they assist in the earlier recovery of kidney function in vascular patients (259). In another Medline search comparing any statin treatment before cardiac surgery, Liakopoulos and colleagues supported Paraskevas’ findings and further discovered that preoperative statin therapy reduced the risk of post-operative AF and shortened ICU and hospital stay (260). Sanders and colleagues examined Cochrane Central Register of Controlled Trials and reported that short-term statin therapy, commenced before or on the day of non-cardiac vascular surgery and continuing for at least 48 h afterward, improved patient outcomes but had no influence on the risk of MI, stroke, renal disease, pain, or length of hospital stay (261). In a prospective randomized trial of 418 consecutive patients undergoing CABG surgery, Ouattara and colleagues concluded that statin therapy was associated with a significant and dose-dependent reduction in adverse cardiovascular events such as heart failure, malignant arrhythmia, and cardiac death after surgery (262). However, they recommended more trials are required including an evaluation of patient tolerance to the therapy (262). Kulik and Ruel in a review of the Medline data (1987 to January 2009) concluded that the benefits of statin use seem to outweigh the risks in CABG surgery, both in the preoperative and postoperative period. In the absence of contraindications, they argued nearly all CABG patients are candidates for life-long statin therapy, which ideally should be started before surgery (263). Chopra and colleagues’ also undertook a meta-analysis and concluded perioperative statin treatment in statin-naive patients reduced atrial fibrillation, MI, and duration of hospital stay (264).

In summary, while statins appear to be well tolerated during surgery, their use has largely come from retrospective and subgroup analysis of large studies from Medline searches. Statins themselves are diverse in their actions and some clinical trials have demonstrated potential benefits while others have not. For example, pravastatin appears to promote risk reduction in the occurrence of new onset diabetes, whereas atorvastatin, rosuvastatin, and simvastatin increase the risk (265). Other questions on whether patients who are already on statin therapy should remain on statin therapy during surgery or those statin-naïve patients should continue after surgery are clinically important to answer (252, 266). Whether statins reduce mortality and morbidity after major surgery or not can only be answered by clear questions and performing properly designed, prospective, randomized, multi-center clinical trials.

### Beta-adrenergic blockers

β-Blockers have a long history of potential beneficial effects in patients with a cardiac risk profile. Some of the benefits include: (1) reducing sympathetic nervous system activity, (2) improving myocardial O_2_ supply/demand ratio from decreased heart rate, systolic blood pressure and myocardial contractility, and (3) having antiarrhythmic properties (267–269). There is also some evidence that beta-blockers may blunt the inflammatory response after injury by reducing the expression of cytokines IL-1, IL-6, and TnF-alpha and CRP (270). Beta-blockers have also been reported in animal and human studies to reduce myocardial ischemia, infarction, and death (269).

#### Non-Cardiac Surgery

Since the late 1990s, multiple retrospective analyses have supported perioperative benefits of β-blockers following surgery (271). However, after a literature search of eleven large databases up to October 2005, Wiesbauer and colleagues concluded that β-blockers did not reduce the incidence of MI, length of hospitalization or mortality (272). They did report there was a trend toward reduced myocardial ischemia and perioperative arrhythmias. On the basis of retrospective analyses, and two small relevant clinical trials, the American College of Cardiology and American Heart Association (ACC/AHA) in 2007 published a set of guidelines recommending perioperative β-blockers for non-cardiac surgery (267, 273). In the following year, a number of groups argued against these guidelines claiming that they were premature and the ACC/AHA should “soften their advocacy” because past trials suffered from a high risk of bias (273).

Moreover, in 2008, the Perioperative Ischemic Evaluation (POISE) trial indicated that long-acting β-blocker metoprolol succinate *increased* mortality, ischemic stroke, hypotension, and bradycardia in patients at high risk of atherosclerotic disease (274). A possible weakness of the POISE trial was its fixed and relatively high-dose of metoprolol that was started shortly before surgery, and this strategy was not consistent with optimal current practice (267, 269). However, despite differences in dose and timing of delivery compared to current practice, the take home message was clear. From retrospective analysis of larger trials and the POISE trial, Devereaux (co-principal investigator of POISE) argued that urgent attention is required to assess the safety and efficacy of perioperative β-blockers (267). The ACC/AHA committee eventually yielded to the mounting pressure and softened their guidelines (269). More recent studies by Angeli and colleagues concluded that β-blockers reduced total mortality in patients who underwent *high-risk non-cardiac surgery* but not lower risk surgery (275). Indeed, the data suggests from low risk of bias trials, an *increase* in all-cause mortality and stroke with β-blocker use (276).

The controversy regarding perioperative β-blockers reached new heights after 2011 with the discovery of scientific misconduct and fabrication in the “Dutch Echocardiographic Cardiac Risk Evaluation Applying Stress Echocardiography (DECREASE)” trials. This was tragic news because the DECREASE family of studies provided much of the original evidence for prophylactic β-blockade use in non-cardiac surgery, and shaped the European Society Cardiology (ESC) Guidelines (250, 269). At the center of the controversy was Don Polderman, chairperson of the ESC guidelines and taskforce on “Pre-operative cardiac risk assessment and perioperative cardiac management in non-cardiac surgery.” Polderman lost his position at the Erasmus Medical Center in Rotterdam for scientific misconduct and the institution released a note of his dismissal on November 16, 2011 stating that he was:

“careless in collecting the data for his research. In one study it was found that he used patient data without written permission, used fictitious data and that two reports were submitted to conferences which included knowingly unreliable data”.

http://www.erasmusmc.nl/corp_home/corp_news-center/2011/2011-11/ontslag.hoogleraar/?lang=en

In 2014, the European Society of Cardiology and European Society of Anesthesiology (ESC/ESA) released joint guidelines with new recommendations stating that β-blockers are not recommended in patients without clinical risk factors, given that the drugs do not decrease the risk of cardiac complications and “may even increase this risk” (276). Despite this warning, the guidelines continue to recommend β-blocker use as reasonable in patients with intermediate- or high-risk myocardial ischemia documented prior to surgery (class IIb, level of evidence C), and for those with three or more risk factors, such as diabetes, heart failure, or coronary artery disease. This “relaxing” of the guidelines appears to ignore the fact that the DECREASE family studies, on which many of the guidelines are based, were deemed “unreliable” and contained “fictitious data” (277). In 2013, Bouri, Francis, Cole, and colleagues argued that initiation of β-blockers in patients undergoing non-cardiac surgery increased the risk of mortality by 27%, potentially resulting in the deaths of as many as 10,000 patients per year in the UK alone (278).

#### Cardiac Surgery

In cardiac surgery, β-blockers are generally recommended to reduce postoperative atrial fibrillation (AF) and cardiovascular ischemic events and have been used for more than 40 years (269, 271, 279). Ogawa and colleagues showed in 136 patients undergoing off-pump CABG that administration of low-dose continuous infusion of ultra short-acting landiolol from the beginning of the operation until postoperative day 2, significantly reduced the incidence of postoperative atrial fibrillation by nearly 50% (19 vs. 37%) and also significantly suppressed systemic inflammation during CABG from a reduced postoperative peak in CRP compared to the non-landiolol group (268, 280). Recently, Blessberger and colleagues concluded after examining 89 randomized controlled trials with 19,211 participants that β-blockers in cardiac surgery can substantially reduce the high burden of supraventricular and ventricular arrhythmias following surgery (276, 281). However, they found that the influence of β-blockers on mortality, AMI, stroke, congestive heart failure, hypotension, and bradycardia in this setting remained unclear (276). In another meta-analysis with more than 100,000 study participants, Bangalore and colleagues warned against use of beta-blockers in post-MI patients because of a possible increase in the risk of heart failure and cardiogenic shock (282).

As with statin use, it appears that the potential benefits of beta-blockers in cardiac or non-cardiac surgery has largely been filtered from retrospective and subgroup analysis of large studies. Well-designed, prospective, randomized clinical trials, with the appropriate statistical power and relevant primary endpoints such as perioperative MI, ischemic stroke, cardiovascular death, and all-cause death are urgently required.

### Calcium-channel blockers

Calcium blockers were originally introduced in surgery to reduce intracellular Ca^2+^ loading and protect against myocardial ischemia and angina pectoris. Prior to 2004, Wijeysundera and colleagues undertook a meta-analysis involving forty-one clinical trials using Ca^2+^ blockers (e.g., amlodipine, nifedipine, nicardipine) and beta-blockers, and concluded that the short-acting Ca^2+^ blockers dihydropyridines were associated with anti-ischemic effects and a trend toward reduced mortality after CABG surgery (283). Over the past decade, the safety and efficacy of Ca^2+^ channel blockers as a group has been controversial and it appears that they have little cardiac benefit in patients undergoing non-cardiac surgery or cardiac surgery (284). In 2008, Kertai and colleagues’ retrospective analysis of a large database showed that dihydropiridines were independently associated with increased 30-day mortality in patients undergoing aortic aneurysm surgery than non-users (285).

Today, there appears to be a growing consensus that Ca^2+^ blockers may be harmful in the perioperative setting in patients undergoing major non-cardiac surgery. Of particular concern is peripheral vasodilation causing a reflex adrenergic activation resulting in an increase in heart rate, which may be associated with myocardial ischemia (284). Thus, in patients with unstable angina, dihydropyridines are contraindicated in the absence of beta-blockade (284). Unfortunately, there are few prospective randomized, prospective, trials that specifically examine hard outcomes associated with Ca^2+^ channel blockers and perioperative hemodynamics, because patients with different hemodynamic profiles may respond with different outcomes (284).

### Where do we stand today?

In those patients already on chronic β-blockers, statins and Ca^2+^ blocker therapies, the general consensus among anesthesiologists and surgeons is to continue their use before and after major surgery (286). With respect to patients not on these drugs and who require major surgery, the data are much less clear. In 2014 Francis, Cole and colleagues argued that the Guideline bodies should retract their recommendations based on fictitious data (278). To this end, Nowbar and colleagues examined 14 such recommendations and dismissed 11 of them based on lack of data or past associations with bias or misconduct. They concluded that there is insufficient evidence to recommend statins, beta-blockers or Ca^2+^ blockers without properly designed trials (250). Bouri, Francis, Cole and colleagues also proposed that: “any remaining enthusiasts might best channel their energy into a further randomized trial which should be designed carefully and conducted honestly” (278). The controversy continues.

## Search for New Therapies from a Systems-Based Approach

What we anticipate seldom occurs; what we *least expect generally happens*.

Benjamin Disraeli (1804-81) *Henrietta Temple*

### Frontline protection begins before the first incision

For a Kuhnian revolution to occur in surgical protection, it is proposed that new drugs and treatment strategies must embrace the CNS control of whole body function. A highly reductionist approach leveled at single drug targets ignores the complexity of biological systems. Reductionism is important in breaking a system into its constituent parts for study, however, it does not do away with the system (287). Thus current practice of identifying, documenting and treating a single perturbation during or following an operation, and then the next defect, and so on down the line, is not working and may result in what US surgeon William C. Shoemaker termed “an uncoordinated and sometimes contradictory therapeutic outcome” (287, 288).

Protection should begin early before the first incision to prevent the body from overshooting its normal homeostatic tolerance limits. Drug targets include the regions of tissue injury, the CNS response to that injury and their systemic manifestations (Table 3). No drug or drug management strategy currently exists to effectively blunt or prevent these stressors and responders to major surgery.

**Table 3:** **Three potential perioperative targets for reducing the stress response to major surgery**.

Target	Stressor, crosstalk, and responder modulation
Tissue injury	Reduce the local tissue damage signals released from the first incision. Dampen pain signals to CNS via modulation of nerve afferents, pain receptors and mediators. Inhibit tissue activation of immune and inflammatory cells, including the production of IL-1, IL-6 and TnF-alpha cytokine and their post-translational pathways. Protect the endothelium and localize the coagulation effects in response to injury.
CNS control	Reduce the brain’s responsiveness to tissue damage signals. Protect the blood brain barrier from becoming leaky and proinflammatory. Reduce activation of the HPA axis and sympathoadrenal system (e.g., cortisol, catecholamines, and vasopressin). Reduce medullary NTS sympathetic discharge in favor of parasympathetic outflow including activation of the anti-inflammatory reflex. Place the body in a mild hibernating-like, hypotensive state. Improve baroreceptor sensitivity and heart rate variability. Optimize arterial resistance and tissue blood flow, including blunting catecholamine-induced changes to splanchnic blood reservoir and circulation to maintain venous return and cardiac output (CO). Reduce gut ischemia and prevent or reduce the gut “cytokine storm”.
Systemic manifestations	Promote cardiovascular-endothelial coupling and induce a high flow, hypotensive, vasodilatory state with maintained tissue O_2_ perfusion. Reduce systemic inflammation and coagulopathy. Protect the gut and liver from “overshooting” their immune functions. Maintain systemic cellular immunity Th1/Th2 cytokine balance and prevent immunosuppression. Reduce whole body energy demand.

### Toward stress-free surgery in the 21st century: A working hypothesis

It is proposed that targeting local tissue injury, the CNS response to that injury and systemic manifestations may improve patient outcomes by reducing surgical trauma and minimizing “secondary-hit” complications from neuroendocrine, inflammatory, coagulation, and metabolic imbalances. The key to maintaining or restoring cellular homeostasis is to provide material exchange between the blood and the tissues. As a working hypothesis, the four pillars of whole body resynchronization during surgical trauma are:

- CNS as *central controller*;
- Heart as *pressure generator*;
- Arterial supply venous capacitance as *pressure/volume regulators*;
- Vascular endothelium as the *systemic integrator*.

**Figure 4:**
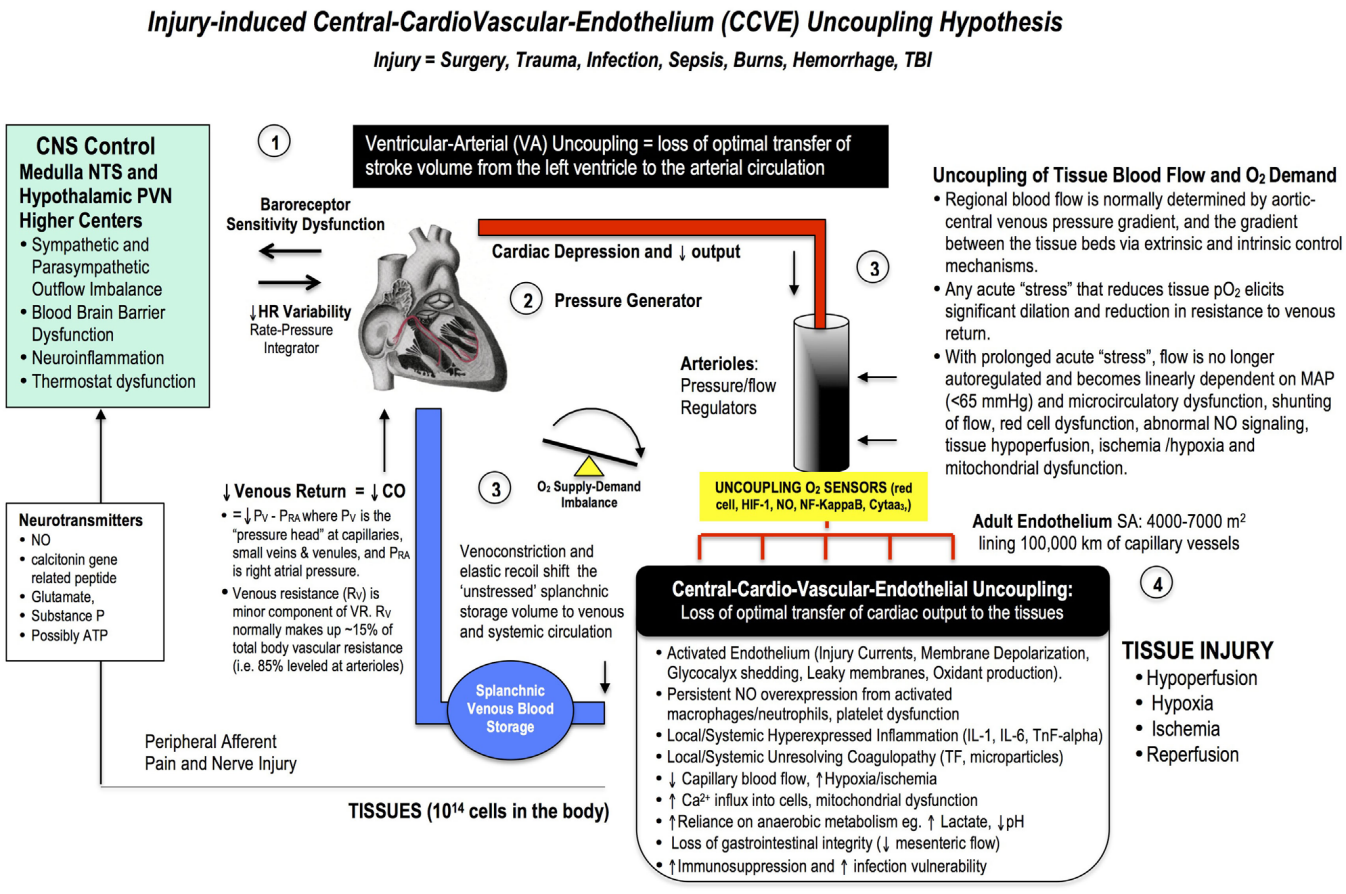
**A broad schematic of the Central-CardioVascular-Endothelium (CCVE) “uncoupling” hypothesis that may be responsible for the high mortality and morbidity after major surgery**. Loss of whole body homeostatic control during surgical trauma may be leveled at: (1) the CNS, (2) the heart, (3) the vascular tree, and (4) the endothelium. There is an urgent need to develop a pharmacological therapy that supports a high flow (maintained cardiac output), hypotensive, vasodilatory state with endothelial protection and tissue oxygenation (287). If central and local control of cardiac output and ventricular-arterial coupling are improved, endothelial and micro-vascular function will be improved and tissue O_2_ delivery will be maintained. An uncoupling is reflected in increased stress hormones, sympathetic discharge, loss of baroreceptor sensitivity, and loss of heart rate variability (229, 230). Impaired sympathetic control and a loss of heart rate variability are two of the strongest predictors of death in critically ill patients (188), and promote a pro-inflammatory state with higher IL-1, IL-6, TnF-alpha, and CRP levels, and coagulopathy. A new whole body therapy is required to bolster the patient’s defense against the trauma of surgery and prevent “secondary hit” complications from ischemic and inflammatory cascades, coagulopathy, multiple organ failure, and immunosuppression.

If imbalances or uncoupling occurs to any of these pillars beyond their normal design tolerances, perioperative complications may arise (Figure 4). This stress-induced mismatch is termed Central-CardioVascular-Endothelium (CCVE) uncoupling. If central and local control of CO and ventricular-arterial coupling are impaired, endothelial and micro-vascular function may be impaired and tissue O_2_ delivery compromised. A stress-induced sympathetic discharge results in loss of heart rate variability and changes to baroreceptor sensitivities, which profoundly impacts CO and hemodynamics and whole body function. If CO is reduced, and the ability of the arterial system to receive blood from the heart is impaired, splanchnic venous capacitance may be diminished and venous return (and CO) will drop further leading to tissue hypoperfusion, endothelium damage, systemic inflammation, and coagulopathy (Figure 4). Maintenance of cardiac preload thus depends on the ability of the CNS to control venous compliance and hence to redistribute blood volume between peripheral organs and the cardiopulmonary compartment.

Maintaining the health of the vascular endothelium is a key to reduce surgical stress because this “organ” is the master integrator and regulator of vascular tone, inflammation and coagulation, vascular permeability, blood fluidity, and lymphatic function (Figures 1–4) (289). Its vast surface area of up to 7000 m^2^ is lined with negatively charged 0.1–1 uM thick glycocalyx mesh of fibril projections made of proteoglycans and glycoproteins (290–294). When injured, the glycocalyx releases syndecan-1, hyaluronic acid, and heparan sulfate into the circulation and the endothelium becomes leaky and damage occurs to underlying tissues (291, 293, 295, 296). Once injured, there is evidence that the glycocalyx can repair itself quickly under the right conditions, which has great significance to surgery and recovery (110, 297). An underlying assumption of the CCVE hypothesis is that if the stress of surgery is controlled, the patient will do the recovery since every cell in the body is programed and working hard in that direction already.

### Perioperative innovation: From natural hibernators to heart surgery

For a large number of problems there will be some animal of choice on which it can be most conveniently studied.

Krogh (298), p. 202

#### Cardiac Surgery

In 1998, the author (GPD) utilized the August Krogh principle and asked: “*Could the human heart in cardiac surgery be pharmacologically manipulated to operate more like a heart of a natural hibernator*?” (25). Natural hibernators are extraordinary animals and can become profoundly hypotensive and hypothermic with up to 98% reductions in body metabolism (25). In this “pilot-light” state, the hibernator does not flood its heart with high potassium, as is standard practice in cardiac surgery today. The objective was to arrest the human heart at its natural resting “polarized” potential of −80 mV by: (1) inhibiting the voltage-dependent Na^+^ fast channels responsible for the phase O upstroke of the action potential (AP) (lidocaine), and (2) simultaneously decreasing the AP duration assisted by opening K^+^_ATP_ channels (adenosine) (191). Magnesium was included to reduce Ca^2+^ entry and protect the heart from ischemia-reperfusion injury and post-operative arrhythmias.

Theoretically, this drug strategy should “flat-line” the heart at its natural “diastolic” membrane potential and confer protection by having fewer channels open, less Na^2+^ and Ca^2+^ loading, less inflammation (from a polarized potential), and less arrhythmias during reanimation (299). What emerged was the world’s first low potassium polarizing adenosine and lidocaine with Mg^2+^ (ALM) cardioplegia (299). Recently an Italian prospective, randomized trial showed that the new cardioplegia was superior to a Buckberg high potassium solution by demonstrating significantly lower perioperative troponin levels, improved post-operative cardiac function (arterial ventricular coupling), 50% less blood transfusions, one full day less in ICU, and two days less in hospital (300). The key to this “polarizing” concept was that ALM at high concentrations arrests the heart, *and at lower concentrations it resuscitates the heart*. What follows are studies involving the lower, *non-arrest* ALM levels to rescue the heart following MI, hemorrhagic shock, cardiac arrest and sepsis, which may have applications to major surgery administered as an IV drip after anesthesia but before the first incision.

#### Possible Applications to Major Surgery

Our first set of *in vivo* rat studies showed that AL infusion administered 5 min before severe regional myocardial ischemia from tying off the left anterior descending coronary artery for 30 min led to 100% survival and a 92% reduction in ventricular arrhythmias compared to 60% deaths in controls (301). We also showed using ^31^P NMR that AL led to improved ATP supply by lowering myocardial demand during insult (302). Our second set of studies showed that ultra-small volumes of an IV bolus of 7.5% NaCl ALM (~3% of shed volume) resuscitated the heart and raised mean arterial pressure (MAP) into the hypotensive range after severe 40–60% blood loss and shock, and it corrected coagulopathy at 60 min in the rat model (303, 304) (305). In 2015, we showed that coagulation correction occurred in 5 min indicating that the coagulopathy was not consumptive because the clotting factors, post-shock platelets, and coagulation pathways were fully operational compared with controls (110, 306). We proposed that small-volume ALM assisted the heart and the body to recover with the blood left in the circulation after severe loss without large volume fluid therapy. In addition, ALM has potent anti-inflammatory properties by reducing the priming and activation of neutrophils (307), reduces TnF-alpha (308) and reduces endothelial damage (306). We have also shown similar protection in the rat model of 8 min asphyxial hypoxia where a small bolus of 0.9% NaCl ALM improved return of spontaneous circulation (ROSC), hemodynamics, coagulation status and survival (108, 309).

Small-volume 7.5% NaCl ALM resuscitation has translated from rat to pig after 75% blood loss (310). Around 2 l of blood was removed from 40 kg pigs and only ~140 ml IV bolus of 7.5% NaCl ALM (~7% return of shed volume) was administered (310). During 60 min hypotensive phase (MAP ~50 mmHg), the ALM group had a 1.8-fold increase in stroke volume, a 34% fall in blood lactate, and a 43% higher O_2_ delivery compared to controls which began decompensate (310). How can 140 ml of ALM fluid increase stroke volume by 1.8 times when added to only ~25% of the animal’s normal circulating blood volume? One possible explanation is that ALM improved the coupling between the heart and arterial supply venous capacitance system, and increased the mean systemic pressure (P_MS_) that was sufficient to increase venous return by 1.8-fold (see Figure 4). After 60 min, shed blood was returned and whole body O_2_ consumption fell, systemic vascular resistance increased 30%, and urine output in the ALM group increased threefold compared with controls (310). Lastly, the hypotensive cardiac rescue potential of 7.5% NaCl ALM in the pig model of 75% blood loss was further demonstrated by Granfeldt and colleagues who showed that a 20 ml bolus (0.5 ml/kg) significantly reduced fluid requirement by 40% to reach a target MAP of 50 mmHg (311). Interestingly, when shed blood was returned a 10 ml bolus of 0.9% NaCl AL (no Mg^2+^) there was a significant drop in whole body O_2_ consumption (27% fall) and improved cardiac and renal function (311).

The ALM drug therapy also appears to be protective against infection. We showed that a bolus and infusion of 0.9% NaCl ALM in the rat model of polymicrobial sepsis elicited a stable, hypotensive state and reduced lung edema compared to controls (312). The therapy also corrected coagulopathy due to the laparotomy itself which may have clinical implications (312). Importantly, IV infusion rates were kept low (1.0 ml/kg/h) to avoid “secondary hit” complications from fluid overload such as cardiovascular and endothelial dysfunction, inflammation and coagulopathy. These low volumes are consistent with human studies by Lamke and colleagues who showed that the basal evaporation rate and typical fluid losses in humans undergoing major abdominal surgery were ~0.5 ml/kg/h (313, 314). Today, high fluid volumes up to 3–4 l are common in major surgery and may amplify the stress response by shocking the body a second time (315–317). In the pig endotoxin model, we also showed that a bolus and infusion of 0.9% NaCl ALM induced a profound hypotensive, vasodilatory state (MAP ~47 mmHg) with maintained CO and tissue oxygenation for 5 h. This state was accompanied by improved ventricular-arterial coupling, a significant reduction in TnF alpha and reduced lung edema compared to LPS controls which began to decompensate (308).

To summarize, our work using a small-volume ALM bolus and infusion “drip” appears to induce a mild hibernating-like state in rat and pig models, and may find clinical utility in protecting the patient against the stress of major surgery. It is possible that an ALM bolus/drip may blunt the sympathetic discharge that accompanies surgical stress and improve the coupling between the CNS support of the cardiovascular system and endothelium to reduce the inflammatory and coagulopathy responses with reduced mortality and morbidity. Clinical safety trials are required to examine the effect of the ALM “drip” (0.25–0.5 ml/kg/h) administered after anesthesia but before the first incision to reduce the stress response of major surgery.

## Concluding Remarks

Major surgery elicits profound changes in the neuroendocrine, metabolic, inflammatory, and immune systems, which collectively constitutes the “stress response.” The stress response is normally self-limiting and resolving. However, during surgical stress, the system can quickly “overshoot” and result in potentially harmful outcomes such as cognitive and cardiac dysfunction, vascular instability, endothelial activation, inflammation, coagulopathy and possibly immunosuppression. Since cardiovascular function is key to a healthy endothelium, it is proposed frontline drugs that improve CNS control of CO and arterial supply venous return functions will help to maintain tissue oxygenation and improve perioperative outcomes. Improved endothelium function may reduce an overexpression of inflammatory, immune and complement discharges and reduce “secondary hit” complications such as SIRS, MODS, and MOF. A systems-based approach to perioperative protection may also find wide utility in treating the critically ill or casualties in prehospital and military environments, and help stabilize the patient during transport to definitive care.

## Author Contributions

GD is the sole contributor to the design, implementation, literature analysis and writing of the manuscript.

## Conflict of Interest Statement

Geoffrey P. Dobson is the sole inventor of the ALM concept for cardioplegia, organ preservation, surgery, infection, and trauma.

## Funding

Internal Research Grant funds (to Geoffrey P. Dobson).
